# Machine learning models based on routine blood and biochemical test data for diagnosis of neurological diseases

**DOI:** 10.1038/s41598-025-09439-4

**Published:** 2025-07-30

**Authors:** Wanshan Ning, Zhicheng Wang, Ying Gu, Lindan Huang, Shuai Liu, Qun Chen, Yunyun Yang, Guolin Hong

**Affiliations:** 1 Department of Laboratory Medicine, Xiamen Key Laboratory of Genetic Testing, School of Medicine, the First Affiliated Hospital of Xiamen University, Xiamen University, Xiamen, 361003 Fujian China; 2 Institute for Clinical Medical Research, School of Medicine, the First Affiliated Hospital of Xiamen University, Xiamen University, Xiamen, 361003 Fujian China; 3 Department of Otolaryngology, School of Medicine, Xiamen University, Xiamen, 361003 Fujian China

**Keywords:** Prediction, Blood routine, Biochemical detection, Machine learning, Nervous system diseases, Diseases, Neurological disorders

## Abstract

Globally, nervous system diseases are the leading cause of disability-adjusted life-years and the second leading cause of mortality in the world. Traditional diagnostic methods for nervous system diseases are expensive. So this study aimed to construct machine learning models using the convenient blood routine and biochemical detection data for diagnosis of nervous system diseases. After the data preprocessing, 25,794 healthy people and 7518 nervous system disease patients with the blood routine and biochemical detection data were utilized for our study. We selected logistic regression, random forest, support vector machine, eXtreme Gradient Boosting (XGBoost), and deep neural network to construct models. Finally, the SHAP algorithm was used to interpret models. The nervous system disease prediction model constructed by XGBoost possessed the best performance (AUC: 0.9782). And the most models of distinguishing various nervous system diseases also had good performance, the model performance of distinguishing neuromyelitis optica from other nervous system diseases was the best (AUC: 0.9095). The model interpretation by SHAP algorithm indicated features from biochemical detection made major contributions to predicting nervous system disease. The present study constructed multiple models using 52 features from the blood routine and biochemical detection data for diagnosis of various nervous system diseases. Meanwhile, distinct hematologic features of various nervous system diseases also were explored. This cost-effective work will benefit more people and assist in diagnosis and prevention of nervous system diseases.

## Introduction

Nervous system diseases, of which the most prevalent are cerebrovascular, neurodegenerative, autoimmune, spinal cord diseases, and so on^1^. In 2021, over 3 billion people worldwide suffered from neurological disorders, affecting 43% of the global population^1^. Globally, Nervous system diseases are the leading cause of disability-adjusted life-years (DALYs, the sum of years of life lost and years lived with disability) and the second leading cause of mortality in the world^2^. Given the absence of cures for numerous nervous system diseases, a thorough comprehension of the risk factors of these nervous system diseases and the development of simple, fast, and cost-effective large-scale population screening methods is in urgent need^3^.

Common strategies for identifying biomarkers associated with central nervous system (CNS) diseases involve diverse modalities of brain imaging^4–6^ and analysis of biomarkers within the cerebrospinal fluid^7,8^. However, these methods are too costly to be used for large-scale disease screening of populations. While nervous system diseases are multifactorial, a majority of them present abnormalities in blood routine and blood biochemical tests^9,10^. For instance, parameters derived from blood routines such as neutrophil-to-lymphocyte ratio, platelet-to-lymphocyte ratio, and monocyte-to-lymphocyte ratio have undergone extensive research and have been established as highly sensitive biomarkers of nervous system diseases^11^. The low-density lipoprotein cholesterol was positively associated with the development of ischemic stroke and strongly inversely associated with intracerebral hemorrhage^10^. Current studies on neurological disease models focus on predicting the risk and prognosis of individual diseases^12–14^. However, there is limited research systematically analyzing distinguishing features between neurological disorders and their unique hematological characteristics.

Significant advancements in biotechnology and health sciences have resulted in the generation of vast quantities of data, including data from medical record systems and laboratory information systems. Doctors often emphasize important abnormal parameters while overlooking a substantial volume of other test data and the interrelationships between laboratory parameters. This tendency may lead to an underestimation of the true efficacy of laboratory data. By integrating multiple tests to analyze potential interactions, we can obtain more valuable information, which is exactly the area where machine learning (ML) excels^15^.

Research on risk prediction models for nervous system diseases is still ongoing, and it holds significant importance for human health to develop a reliable model for monitoring and preventing their occurrence. Yu et al. developed a model integrating cerebral fluid data with non-motor clinical features, such as olactory inormation, to distinguish between healthy controls (*n* = 138) and Parkinson’s disease (*n* = 290) subjects^16^. Omranid et al. identified predictive markers through AI analysis of blood transcriptomics, achieving a 97% accuracy in diagnosing multiple sclerosis^17^. Alexey S. Kononikhin et al. used human plasma protein quantitative mass spectrometry and machine learning to identify 31 proteins crucial for the differentiation of Alzheimer’s disease^18^. These studies have significantly enriched the methods for diagnosing and predicting neurological diseases, reducing the costs of large-scale population screenings to some extent, and demonstrated the feasibility of using hematological indicators for diagnosing neurological disorders. However, most diagnostic and predictive models for neurological diseases have been developed based on blood omics data, imaging data, or cerebrospinal fluid biomarkers, with few studies exploring the use of more accessible and cost-effective routine blood tests and biochemical markers for predicting neurological diseases. Tasci et al. utilized CBC data, including complete blood count with differential (CBC WBC DIFF) and numerical-CBC WBC DIFF scattergrams (which provide information on cell size and visualize it in a two-dimensional scatter plot, clearly displaying monocytes, neutrophils, eosinophils, and lymphocytes), in combination with a deep learning algorithm to aid in the diagnosis of schizophrenia (SZ)^19^. This approach achieved a remarkably high detection accuracy for SZ, inspiring us to explore the use of routine blood tests for diagnosing neurological diseases. Moreover, earlier studies have primarily focused on the prediction and diagnosis of specific nervous system diseases, so there is a lack of comprehensive investigation assessing the prediction of neurological diseases.

Traditional diagnostic methods for neurological disorders are expensive. So in this study, we obtained blood routine and blood biochemical data from patients with nervous system diseases and corresponding healthy populations from the First Affiliated Hospital of Xiamen University. We compared multiple ML algorithms and conducted comprehensive feature interpretation to explore risk factors for nervous system diseases and constructed robust models for assessing the risk of developing nervous system diseases and validate their usability. Our research shows that establishing predictive models for neurological disorders using readily available blood routine and blood biochemistry data has the potential to facilitate early diagnosis and low-cost population screening for these conditions.

## Methods

### Data collection and processing

**Fig. 1:**
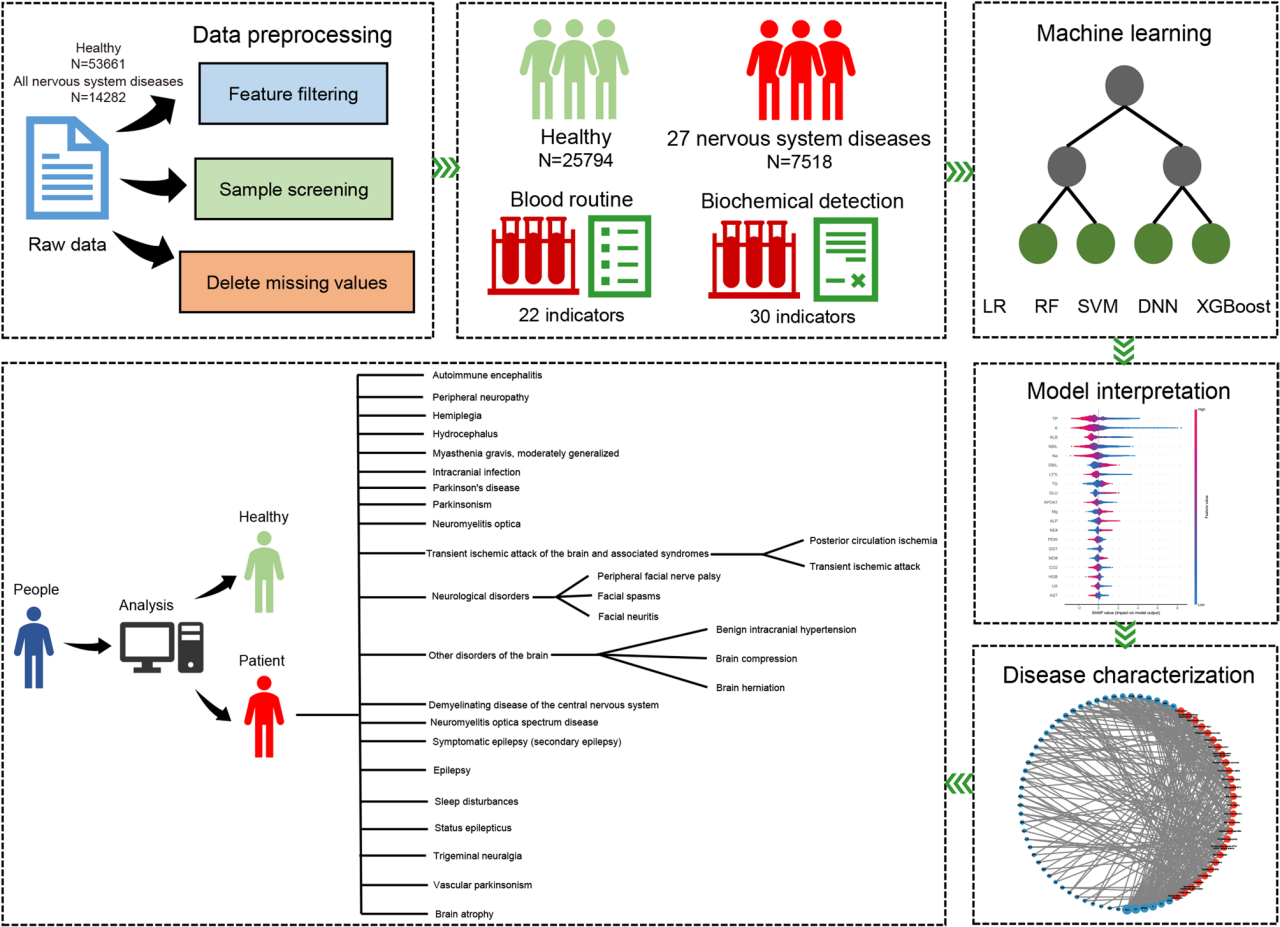
The flow chart of this study.

All the raw data we collected came from inpatients in the Department of Neurology and healthy people who had physical examinations in the First Affiliated Hospital of Xiamen University between 2018 and 2023. These data were from the hospital information system. The diagnostic information and blood routine biochemical test data of these individuals were integrated. For all patients, we screened the blood routine and biochemical test data from the first test after hospitalization as features for the construction of models, while for healthy people, we selected the blood routine and biochemical test data from the first physical examination every year as features. Because too many missing values may affect the prediction accuracy, we removed the features with a missing value ratio greater than 50%, and finally screened out 22 features from the blood routine and 30 features from the biochemical test data (Supplementary Tables 1 and 2). Diagnostic information for all patients was determined according to The International Statistical Classification of Diseases and Related Health Problems 10th Revision (ICD-10). To ensure that the sample size for each nervous system disease was sufficient, we removed nervous system diseases with fewer than 100 samples. Subsequently, to ensure the authenticity of the data, all samples with missing values were removed. In the end, 25,794 healthy people and 7518 patients with nervous system disease were used to construct our models (Fig. 1; Table 1). These data were randomly divided into a training set (70%) and a validation set (30%).

**Table 1:** Data distribution of diseases.

ICD-10 disease code	Disease	Number
G04.801	Autoimmune encephalitis	122
G06.006	Intracranial infection	195
G20.x00	Parkinson’s disease	115
G20.x03	Parkinsonism	447
G21.400	Vascular parkinsonism	110
G31.902	Brain atrophy	537
G36.000	Neuromyelitis optica	134
G36.000 × 002	Neuromyelitis optica spectrum disease	317
G37.900	Demyelinating disease of the central nervous system	123
G40.800 × 004	Symptomatic epilepsy (secondary epilepsy)	191
G40.900	Epilepsy	483
G40.901	Epilepsy	148
G41.900	Status epilepticus	107
G45.004	Posterior circulation ischemia	1259
G45.900 × 001	Transient ischemic attack	110
G47.900	Sleep disturbances	198
G50.000	Trigeminal neuralgia	211
G51.003	Peripheral facial nerve palsy	392
G51.301	Facial spasms	275
G51.800 × 002	Facial neuritis	155
G62.901	Peripheral neuropathy	201
G70.004	Myasthenia gravis, moderately generalized	121
G81.900	Hemiplegia	189
G91.900	Hydrocephalus	569
G93.200	Benign intracranial hypertension	193
G93.500	Brain compression	293
G93.501	Brain herniation	323

ICD-10: The International Statistical Classification of Diseases and Related Health Problems 10th Revision.

### Machine learning methods

Logistic regression (LR), also known as logistic regression analysis, is a generalized linear regression analysis model, which is often used in data mining, automatic disease diagnosis, economic forecasting, and other fields. Logistic regression estimates the probability of an event occurring based on a given dataset of independent variables, and since the outcome is a probability, the dependent variable ranges between 0 and 1. Random forest (RF) is a classifier with many decision trees, which can be used to deal with classification and regression problems, as well as for dimensionality reduction problems. It also has a good tolerance for outliers and noise and has better prediction and classification performance than decision trees. Support vector machine (SVM) is a kind of generalized linear classifier that classifies data binarily according to supervised learning, and its decision boundary is the maximum margin hyperplane solved by the learning sample. eXtreme Gradient Boosting (XGBoost) is an algorithm or engineering implementation based on the Gradient Boosting Decision Tree (GBDT). XGBoost is efficient, flexible, and lightweight, and has been widely used in data mining, recommender systems, and other fields. The deep neural network (DNN) is a framework for deep learning, that is a neural network with at least one hidden layer. Similar to shallow neural networks, deep neural networks can also provide modeling for complex nonlinear systems, but the extra layers provide a higher level of abstraction for the model, thus improving the model’s capabilities. For comparing the performance of different machine learning methods, we selected LR, RF, SVM, XGBoost, and DNN to construct the model^20–24^. The LR, RF, and SVM were used through scikit-learn (version 1.3.0), XGBoost was used through the xgboost package (version 2.0.2), and the DNN by tensorflow (version 2.0.2) in python.

All features were standardized prior to being used for model training. During model training, we addressed the issue of class imbalance by adjusting the class_weight parameter for LR, RF, SVM, and DNN algorithms. For LR, the class_weight mechanism increases the loss weight for minority class samples during cross-entropy loss calculation, ensuring the model pays more attention to these samples. In RF, class_weight adjusts the sample weights during tree construction, balancing the computation of information gain or Gini impurity. For SVM, the class_weight parameter assigns different penalty weights to samples of different classes in the optimization objective, amplifying the influence of minority class support vectors. In DNN, class_weight assigns weights to samples of different classes, making minority class samples more significant in loss calculations. For the XGBoost algorithm, we handled class imbalance by adjusting the scale_pos_weight parameter. This parameter modifies the gradient and Hessian calculations in the objective function, assigning a weight factor to positive samples, thereby altering the influence of positive and negative samples on model optimization.

For all five machine learning algorithms, we used grid search cross-validation (CV) combined with manual fine-tuning to identify the optimal parameters and mitigate overfitting risks. In the hyperparameter optimization process for the five algorithms, we aimed to strike a balance between computational cost and search comprehensiveness. Specifically, we designed representative and reasonable parameter grids based on the characteristics of each algorithm, ensuring robustness and reliability of the results through CV. Meanwhile, we employed parallel computing techniques to further enhance optimization efficiency. Since evaluating each hyperparameter combination typically involves extensive training and validation steps, especially during CV, the computational demand escalates rapidly. To accelerate this process, we utilized multi-core processors for parallel computation, ensuring simultaneous evaluations of multiple hyperparameter combinations, thereby significantly reducing overall optimization time. Additionally, we allocated resources judiciously in parallel computing to maintain efficiency without overloading system resources or causing performance degradation. By adopting this approach, we effectively reduced computational costs while ensuring comprehensive optimization, thereby enhancing the feasibility and efficiency of our experiments. Taking LR and SVM as examples, we optimized the following hyperparameters in detail. For LR, we focused on tuning the penalty, selecting between commonly used L1 and L2 regularization types. The parameter C was set to five values (0.5, 1, 2, 3, and 4) ranging from 0.5 (strong regularization) to 4 (weak regularization), covering typical ranges. The solver was chosen from five mainstream optimization algorithms (e.g., liblinear, saga, lbfgs). Initial experiments revealed that certain solvers were incompatible with specific parameter combinations (e.g., L1 regularization), leading us to narrow the solver range in subsequent steps to reduce computational load. For SVM, we optimized the following hyperparameters: C was set between 0.1 and 100, covering a wide range from strong to weak regularization, with the final values being 0.1, 1, 10, and 100. The kernel was limited to linear and rbf to avoid the computationally expensive poly kernel. The gamma parameter was explored within a range from 0.001 to 1, along with the scale option, to investigate the impact of kernel functions at different scales. This kernel selection strategy avoided expensive computations in high-dimensional data while balancing model expressiveness and efficiency by adjusting the gamma and C ranges. Using this methodology, we applied similar balanced strategies across all algorithms for hyperparameter optimization. On the one hand, we ensured search comprehensiveness by covering key parameters that could affect model performance. On the other hand, we controlled computational costs by reasonably limiting the search space, reducing invalid combinations, and adopting appropriate CV settings. We employed 5-fold CV, a robust evaluation method that divides the dataset into five mutually exclusive subsets. In each iteration, one subset serves as the validation set, while the remaining four subsets are used for training. This process is repeated five times, ensuring that each subset is used as the validation set exactly once. The model is trained in each iteration, and the validation performance is recorded. The average performance across the five iterations is calculated as the final performance metric. This approach maximizes data utilization, effectively assesses model generalizability, and reduces the impact of randomness from a single split. We further divided the dataset chronologically to validate the model’s generalization capability. Data from 2018 to 2021 were split into training and validation sets in a 7:3 ratio, while data from 2022 to 2023 were used exclusively as a test set.

After conducting the above series of grid search attempts, we finalized the parameter adjustments for all machine learning methods as follows. For LR, we optimized the C, penalty, and solver parameters. For instance, reducing the C value and using L2 regularization helped mitigate overfitting. For RF, the min_samples_leaf and n_estimators parameters were fine-tuned. For SVM, we adjusted the C, kernel, and gamma parameters. In XGBoost, we optimized colsample_bytree, gamma, learning_rate, max_depth, n_estimators, and subsample. To avoid overfitting, we decreased max_depth, reduced colsample_bytree, and increased n_estimators. For DNN, we adjusted the activation, number of layers, and number of neurons per layer parameters, and applied L2 regularization to the Dense layers to reduce overfitting risks. The DNN architecture used in this study consists of a four-layer structure, detailed as follows: The first layer is the input layer, with the number of neurons matching the number of input features. The second layer is a hidden layer with 64 neurons and utilizes the ReLU activation function. The third layer is also a hidden layer with 64 neurons and employs the ReLU activation function. The fourth layer is the output layer, containing a single neuron with a Sigmoid activation function, designed for binary classification tasks.

### Model performance evaluation

The model was trained in the training set and then verified in the validation set. Sensitivity (Sn), specificity (Sp), positive predictive value (PPV), negative predictive value (NPV), F1 score, matthews correlation coefficient (MCC), and accuracy (Acc) were utilized for model performance evaluation. Their formulas are shown below^25–27^:

$$\:\text{Sn}\text{}\text{=}\text{}\frac{\text{TP}}{\text{TP}\text{\:+\:FN}}$$

$$\:\text{S}\text{p\:}\text{=}\text{}\frac{\text{T}\text{N}}{\text{T}\text{N\:+\:FP}}$$

$$\:\text{PPV\:}\text{=}\text{}\frac{\text{T}\text{P}}{\text{T}\text{P\:+\:FP}}$$

$$\:\text{NPV\:}\text{=}\text{}\frac{\text{T}\text{N}}{\text{T}\text{N\:+\:FN}}$$

$$\:\text{Acc\:}\text{=}\text{}\frac{\text{T}\text{P\:+\:TN}}{\text{T}\text{P\:+\:FN\:+\:TN\:+\:FP\:}}$$

$$\:\text{F1\:score\:}\text{=}\text{}\frac{\text{2}\text{T}\text{P}}{\text{2}\text{T}\text{P\:+\:FN\:+\:FP\:}}$$

$$\:{\text{MCC}}\:{\text{ = }}\frac{{{\text{TP }} \times \:{\text{TN}}\:{\text{ - }}\:{\text{FP}}\: \times \:{\text{FN}}}}{{\sqrt {({\text{TP}}\:{\text{ + }}\:{\text{FP}})({\text{TP}}\:{\text{ + }}\:{\text{FN}})({\text{TN}}\:{\text{ + }}\:{\text{FP}})({\text{TN}}\:{\text{ + }}\:{\text{FN}})} }}$$

TP, TN, FP, and FN represent true positive, true negative, false positive, and false negative separately. Meanwhile, we also made use of the area under the curve (AUC) of the receiver operating characteristics curve (ROC) to evaluate the model performance comprehensively and select the best algorithm (Supplementary Fig. 1).

### Model interpretation

Machine learning makes it difficult to explain the contribution of each feature due to its black-box principle, so the SHAP algorithm was introduced in this study. The SHAP algorithm assigns a SHAP value to each feature, which is used to explain the impact of the feature on the predictive model^28^. The SHAP value of each feature was computed by the shap python package (version 0.44.0).

## Results

### Nervous system disease prediction model construction

**Fig. 2:**
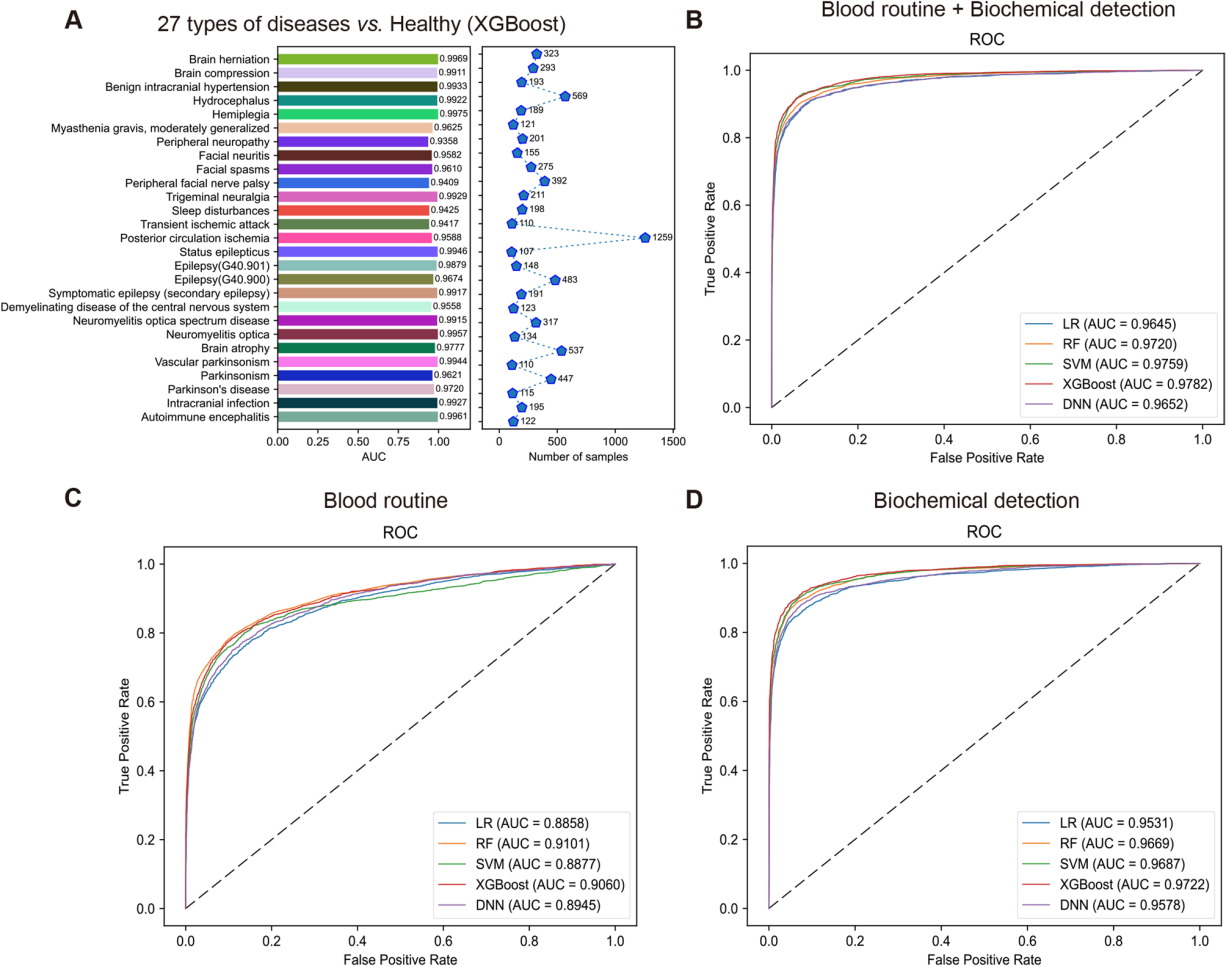
Construction of nervous system disease prediction model using clinical blood samples. (**A**) The AUC of 27 nervous system disease prediction models. ROC curves of five machine learning methods using different data. (**B**) Blood routine combined with biochemical detection. (**C**) Blood routine. (**D**) Biochemical detection.

To ensure the accuracy of our prediction models, the number of various nervous system diseases was all over 100 (Table 1 and Supplementary Data 1). The male-to-female ratio between healthy people and nervous system disease patients was similar, all close to 1:1. The number of healthy people for 40–60 years old and nervous system disease patients for 60–80 years old were the most population, 12,828 and 3233 respectively (Supplementary Fig. 2). Subsequently, we chose five machine learning methods (LR, RF, SVM, XGBoost, and DNN) and utilized 22 features from blood routine and 30 features from biochemical detection to construct the nervous system disease prediction models. The results showed the comprehensive performance of XGBoost was the best (AUC: 0.9782; Acc: 0.9507; Sn: 0.8481; Sp: 0.9805; PPV: 0.9267; NPV: 0.9569; MCC: 0.8556; F1 score: 0.8856). Meanwhile, we also attempted to construct the models only using blood routine or biochemical detection data. We found the model performance of the blood routine combined with biochemical detection was the best (Fig. 2B-D; Table 2). Considering the imbalance for sample number among 27 nervous system diseases, we also used each nervous system disease to construct 27 models. The AUC of these models were all beyond 0.9, the highest one reached 0.9975 (Fig. 2A and Supplementary Fig. 3). These models all showed nice performance and robustness (Table 2).

**Table 2:** Model performance evaluation results (Nervous system diseases vs. Healthy, XGBoost).

Model	AUC	Acc	Sn	Sp	PPV	NPV	MCC	F1 score
Nervous system disease	0.9782	0.9507	0.8481	0.9805	0.9267	0.9569	0.8556	0.8856
Autoimmune encephalitis	0.9961	0.9979	0.6667	0.9997	0.9333	0.9982	0.7879	0.7778
Intracranial infection	0.9927	0.9968	0.6271	0.9996	0.9250	0.9972	0.7602	0.7475
Parkinson’s disease	0.9720	0.9972	0.4878	0.9999	0.9524	0.9973	0.6805	0.6452
Parkinsonism	0.9621	0.9883	0.4559	0.9977	0.7750	0.9905	0.5892	0.5741
Vascular parkinsonism	0.9944	0.9979	0.5161	0.9999	0.9412	0.9981	0.6962	0.6667
Brain atrophy	0.9777	0.9919	0.6867	0.9984	0.9048	0.9933	0.7844	0.7808
Neuromyelitis optica	0.9957	0.9978	0.6000	0.9999	0.9600	0.9979	0.7580	0.7385
Neuromyelitis optica spectrum disease	0.9915	0.9943	0.5938	0.9992	0.9048	0.9950	0.7304	0.7170
Demyelinating disease of the central nervous system	0.9558	0.9952	0.1667	0.9997	0.7778	0.9955	0.3587	0.2745
Symptomatic epilepsy (secondary epilepsy)	0.9917	0.9962	0.5439	0.9995	0.8857	0.9966	0.6924	0.6739
Epilepsy (G40.900)	0.9674	0.9910	0.6149	0.9982	0.8667	0.9927	0.7258	0.7194
Epilepsy (G40.901)	0.9879	0.9970	0.5000	0.9999	0.9565	0.9972	0.6904	0.6567
Status epilepticus	0.9946	0.9981	0.5333	0.9999	0.9412	0.9982	0.7077	0.6809
Posterior circulation ischemia	0.9588	0.9759	0.5990	0.9948	0.8535	0.9801	0.7035	0.7039
Transient ischemic attack	0.9417	0.9964	0.1935	0.9996	0.6667	0.9968	0.3580	0.3000
Sleep disturbances	0.9425	0.9932	0.1613	0.9999	0.9091	0.9933	0.3814	0.2740
Trigeminal neuralgia	0.9929	0.9977	0.7222	0.9996	0.9286	0.9981	0.8179	0.8125
Peripheral facial nerve palsy	0.9409	0.9882	0.3529	0.9979	0.7241	0.9901	0.5006	0.4746
Facial spasms	0.9610	0.9955	0.6145	0.9996	0.9444	0.9959	0.7599	0.7445
Facial neuritis	0.9582	0.9943	0.1739	0.9992	0.5714	0.9951	0.3132	0.2667
Peripheral neuropathy	0.9358	0.9929	0.1273	0.9991	0.5000	0.9938	0.2498	0.2029
Myasthenia gravis, moderately generalized	0.9625	0.9960	0.2927	0.9997	0.8571	0.9963	0.4996	0.4364
Hemiplegia	0.9975	0.9973	0.7091	0.9994	0.8864	0.9979	0.7915	0.7879
Hydrocephalus	0.9922	0.9939	0.8218	0.9978	0.8938	0.9960	0.8540	0.8563
Benign intracranial hypertension	0.9933	0.9971	0.6721	0.9996	0.9318	0.9974	0.7901	0.7810
Brain compression	0.9911	0.9953	0.6632	0.9994	0.9265	0.9959	0.7817	0.7730
Brain herniation	0.9969	0.9972	0.8431	0.9992	0.9348	0.9979	0.8864	0.8866

### Classification of various nervous system diseases

**Fig. 3:**
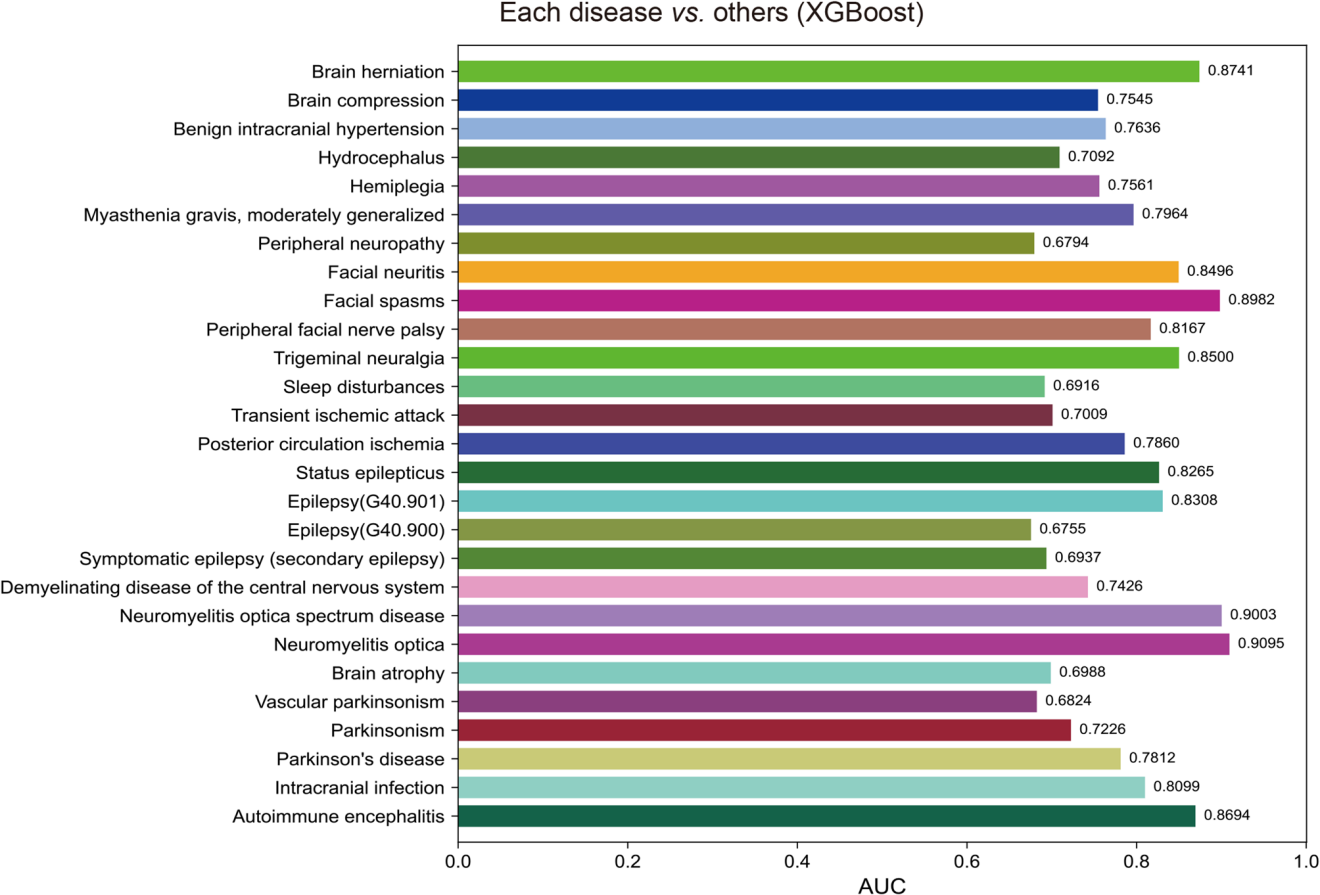
The AUC of 27 models distinguishes a kind of nervous system disease from others.

To further subdivide various nervous system diseases, we constructed 27 models distinguishing a kind of nervous system disease from other nervous system diseases, such as distinguishing brain herniation from other nervous system diseases. The XGBoost was selected to construct models because of its good performance. The results showed the AUC of these models ranged from 0.6755 to 0.9095. Surprisingly, the model performance of distinguishing neuromyelitis optica (NMO) from other nervous system diseases was the best. NMO is an acute or subacute demyelinating lesion in which the optic nerve and spinal cord are simultaneously or sequentially affected. The diagnosis of NMO primarily depends on Magnetic Resonance Imaging (MRI) and serum NMO-IgG detection, not the blood routine and biochemical detection. These results indicated these models could help doctors well distinguish different nervous system diseases (Fig. 3).

### Analysis of nervous system disease-specific indicators

**Fig. 4:**
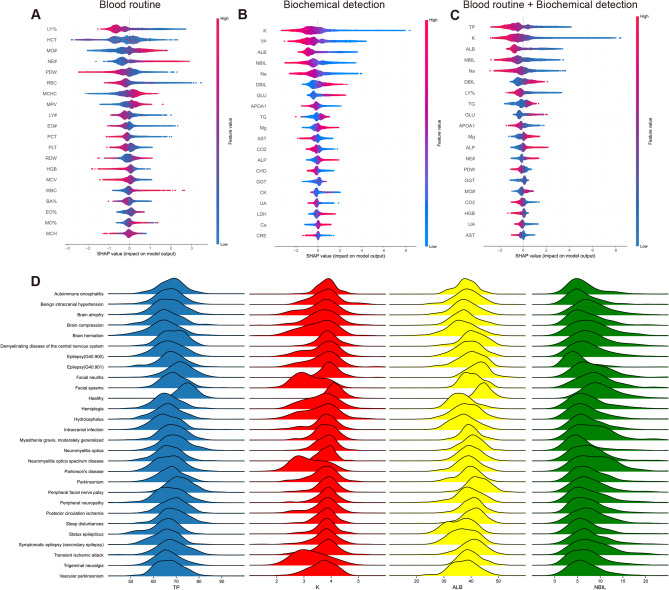
The top 20 features for the nervous system disease prediction model using different data. (**A**) Blood routine. (**B**) Biochemical detection. (**C**) Blood routine combined with biochemical detection. The red represents a high value, and the blue represents a low value. If the SHAP value is positive, it represents the positive effect of the feature on the model, and vice versa. All features are listed in order of importance from top to bottom. (**D**) The joyplot of numerical distributions of TP, K, ALB, and NBIL among various nervous system diseases and healthy people.

To help us better understand the contributions of 52 features for the nervous system disease prediction model and find the nervous system disease-specific indicators, we used the SHAP algorithm to compute the contribution degree of each feature. For the constructed model only utilizing the blood routine, the top 10 features were lymphocyte percentage (LY%), hematocrit (HCT), absolute value of monocyte (MO#), absolute value of neutrophil (NE#), platelet distribution width (PDW), red blood cell count (RBC), mean erythrocyte hemoglobin concentration (MCHC), mean platelet volume (MPV), absolute value of lymphocytes (LY#), and absolute value of eosinophil (EO#) (Fig. 4A). For the constructed model only utilizing the biochemical detection data, the top 10 features were potassium (K), total protein (TP), albumin (ALB), indirect bilirubin (NBIL), sodium (Na), direct bilirubin (DBIL), glucose (GLU), Apolipoprotein A1 (APOA1), triglycerides (TG), and magnesium (Mg) (Fig. 4B). Interestingly, for the constructed model utilizing the blood routine combined with biochemical detection, only one feature from the blood routine, LY%, was one of the top 10 features (Fig. 4C). These results indicated features from biochemical detection made major contributions to predicting nervous system disease (Fig. 4D). To further validate the importance of these features, we also calculated the top 20 features ranked by four other machine learning methods. As shown in the results, although the feature rankings varied slightly across methods, there was substantial overlap among the top 20 features. This indicates that our model interpretation method demonstrates good stability (Supplementary Data 2).

To verify whether the performance of the XGBoost was affected by redundant features, we constructed the model to distinguish nervous system disease patients from healthy individuals using only the top 10 features ranked by the SHAP algorithm (Fig. 4C). The results showed that the model constructed with the top 10 ranked features performed similarly to the model built with all 52 features (Supplementary Fig. 4).

### Analysis of characteristic indicators of discrimination between various nervous system diseases

**Fig. 5:**
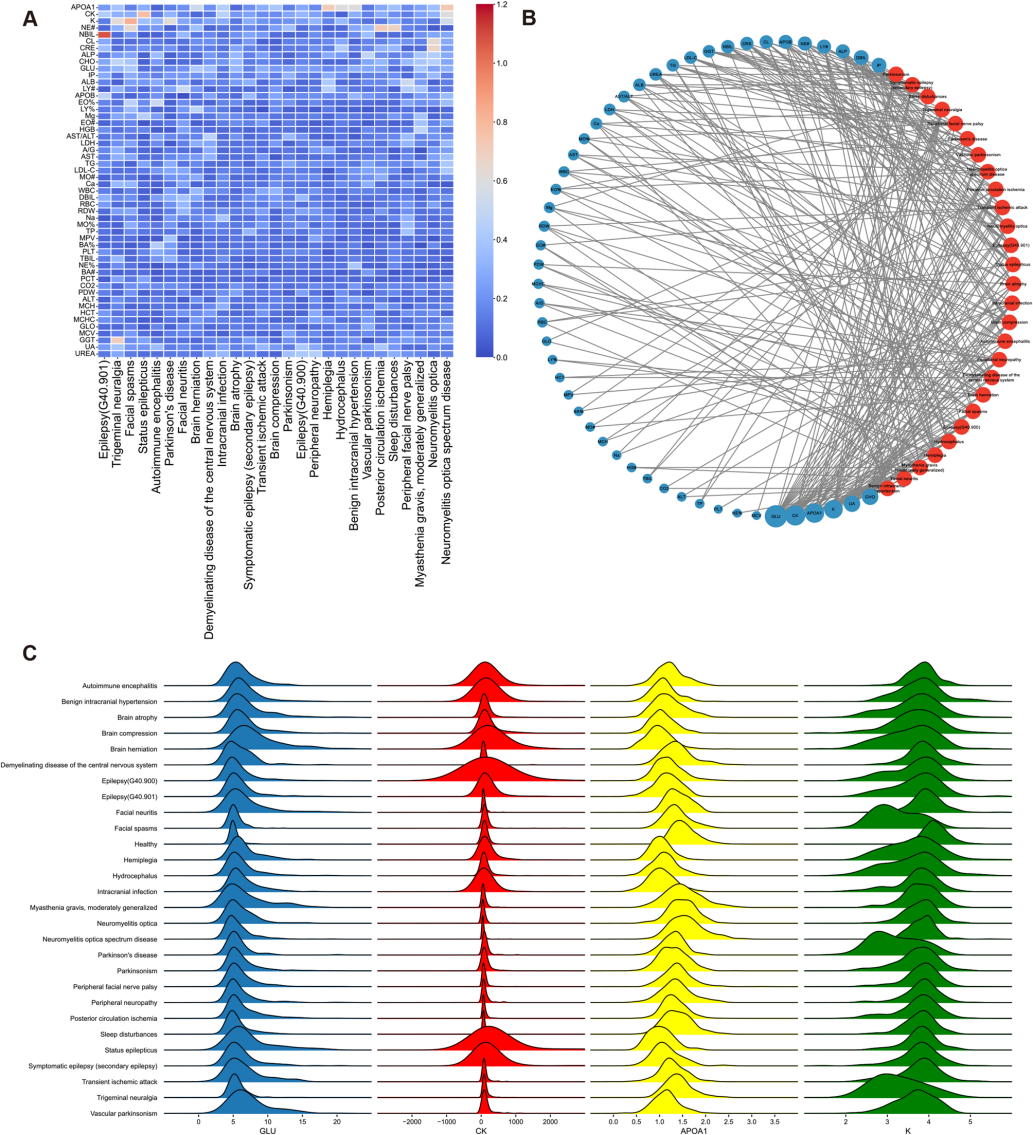
Analysis of specific indicators for differentiation between different nervous system diseases. (**A**) The heatmap displaying SHAP values of 52 features for each disease differentiation model. The positive SHAP value is added to the absolute value of the negative SHAP value to form the final SHAP value to be displayed. (**B**) The network showing the intersection top 10 features among different disease differentiation models. The red circles represent various nervous system diseases, and the blue circles represent various features. The larger the blue circle, the more the intersection features. (**C**) The joyplot of numerical distributions of GLU, CK, APOA1, and K among various nervous system diseases and healthy people.

After exploring nervous system disease-specific indicators, we also hoped to further explore characteristic indicators of discrimination between various nervous system diseases. Then, we displayed the SHAP value of each feature through a heatmap. Rows and columns were clustered separately, and the more similar the features or diseases, the closer they were. We found that every nervous system disease had distinctive characteristics (Fig. 5A). At the same time, a network displaying the intersection features among various nervous system diseases showed that GLU, creatine kinase (CK), APOA1, and K were the top 4 features subdividing various nervous system diseases (Fig. 5B). Elevated GLU is often associated with diabetes, but we found that GLU could also be used to distinguish between different nervous system diseases. APOA1 is the main protein component of high-density lipoprotein (HDL) in plasma and is commonly used as a biomarker to predict cardiovascular disease. And as we can see in our results, it also has great potential for predicting various nervous system diseases. The numerical distributions of the top 4 features among various nervous system diseases and healthy people were different (Fig. 5C). The results proved our models were reliable.

## Discussion

Diagnosing nervous system diseases relies on a comprehensive evaluation of medical history, physical examinations, and imaging studies. Nevertheless, neurological symptoms and signs can lack specificity, with clinical features frequently overlapping. Particularly in the early stages of many nervous system diseases, symptoms may be nonspecific^29,30^. Clinical reliance on imaging techniques such as Computed Tomography (CT) and MRI is common, yet economically underdeveloped regions often struggle to afford them, and they are not suitable for population-based screening as well as long-term monitoring^31,32^. Additionally, the sensitivity of imaging detection is low in the early stage of nervous system diseases. When patients seek medical care, doctors usually conduct blood routine and blood biochemical tests as part of their evaluation. Given the simplicity, accessibility, and low cost of these medical test data, we attempted to use blood routine and blood biochemical tests to establish disease prediction models.

In this work, the blood routine and blood biochemical data of 25,794 healthy people and 7518 patients with nervous system disease from the First Affiliated Hospital of Xiamen University were analyzed. We selected 5 kinds of ML methods (LR, RF, SVM, XGBoost, and DNN) and a total of 22 features from the blood routine and 30 features from blood biochemical tests were selected to construct a prediction model for nervous system diseases. The model demonstrated a robust predictive performance. Subsequently, we attempted to construct predictive models to distinguish among 27 common nervous system diseases. All these 27 prediction models can discriminate among multiple nervous system diseases, with particularly notable performance in distinguishing NMO (AUC = 0.9095) from other nervous system diseases.

We have developed a multivariate predictive model utilizing blood routine and blood biochemical tests. This model holds promise as a reliable method for early diagnosis and population screening of nervous system diseases. Neuroscientist Jonathan Kipnis’ work has revealed the dural sinuses as the interface for neural and immune interaction, facilitating communication between the CNS and the immune system^33^. Studies have shown that abnormal B-lymphocyte counts and immune responses in the peripheral blood may play important roles in the development of Parkinson’s disease^34^. Changes in the blood-brain barrier can facilitate the entry of peripheral blood lymphocytes into the CNS, potentially involving them in the pathogenesis of nervous system diseases. As Alzheimer’s disease and Parkinson’s disease progress, the peripheral blood lymphocyte profiles of patients undergo notable changes, affirming the significance of LY% as a predictive feature in the model^35^.

The model in this study incorporated K, Na, and Mg concentrations in blood tests by training. Sodium is the primary extracellular cation, while K and Mg are the primary intracellular cations. Metal homeostasis is critical to normal neurophysiological activity. For example, K is crucial for regulating neuronal excitability by maintaining resting membrane potential and influencing action potential dynamics. K channels help balance potassium distribution, stabilizing the neuronal environment and preventing overexcitability^36^. It is noteworthy that indicators reflecting liver function, such as DBIL, NBIL, TP, and ALB, along with markers associated with liver glucose and lipid metabolism, such as GLU and APOA1, play significant roles in predicting nervous system diseases. This suggests a close association between the occurrence of nervous system diseases and liver function through metabolic homeostasis^37^. Albumin also plays a significant role in maintaining blood-brain barrier integrity, and is particularly effective in neuroprotective contexts^38^. Subsequently, we further explored characteristic discriminative indicators among various nervous system diseases. We found that the numerical distribution of GLU, CK, APOA1, and K could serve as important features for distinguishing different nervous system diseases. Hyperglycemia increases oxidative stress and matrix metalloproteinase-9 (MMP-9) activity and leads to brain-blood-barrier dysfunction^39^. Creatine kinase is part of the phosphagen kinase family of guanidine kinases, whose primary function is to help in Adenosine 5’-triphosphate hydrolysis^40^. Patients with nervous system diseases such as amyotrophic lateral sclerosis, stroke, and epileptic seizures often exhibit elevated levels of CK in their peripheral blood. APOA1, the primary apolipoprotein component of HDL, plays a direct role in cholesterol efflux in the brain and is strongly linked to atherosclerosis and dementia^41–44^. Higher serum APOA1 level is also correlated with reduced blood-brain barrier injury and a lower risk of neuronal degeneration^45^.

While earlier research has predominantly centered on diagnosing specific neurological conditions such as Parkinson’s disease, epilepsy, Transient Ischemic Attack, and Myasthenia gravis, the development of comprehensive diagnostic models covering a broad spectrum of neurological diseases has been less common^16,46–48^. Simon Lam et al. constructed a multinomial model with demographic data, blood and urine test results, and cognitive assessments from 1,223 UK Biobank participants, achieving an 88.3% accuracy in predicting Alzheimer’s disease, Parkinson’s disease, motor neuron disease, and myasthenia gravis^49^. In our study, we performed an extensive analysis of 27 prevalent neurological diseases, developing diagnostic models through XGBoost that achieved a 95% accuracy rate. Additionally, our comprehensive approach in constructing the model included an analysis of distinctions between different neurological diseases, thereby providing physicians with improved diagnostic differentiation.

Our findings confirm that by applying machine learning to data from neurological patients, it is possible to diagnose various types of nervous system diseases using blood routine and biochemical tests, as well as differentiate between these diseases. Our ML model is capable of extracting subtle diagnostic information from blood test results, data that even the most experienced clinicians may find difficult to detect. We believe that this approach holds the potential to improve traditional diagnostic procedures, and open up a new pathway for diagnosing these diseases. It facilitates large-scale screening of at-risk populations, reduces testing costs, and supports early treatment. To further support real-world application, we added a case study to illustrate the clinical utility of our model. A 63-year-old female patient (Patient A) presented with dizziness, memory decline, and gait instability. Her blood test showed decreased K and APOA1 levels. Our model predicted a high risk (89%) of posterior circulation ischemia. This was later confirmed by MRI and Magnetic Resonance Angiography (MRA). SHAP analysis highlighted the same two features as major contributors to the prediction. This case demonstrates both the practical applicability of our model and the interpretability afforded by SHAP in guiding clinical decision-making. We also added details on model training time and hardware requirements. The XGBoost model was trained on a device with an 11th Gen Intel(R) Core(TM) i7-11800 H @ 2.30 GHz Central Processing Unit (CPU) and 16GB of Random Access Memory (RAM). The average training time was within 5 min, indicating good scalability for large-scale population screening and real-time clinical deployment. Furthermore, we expanded our discussion on the use of SHAP values. These values help clinicians interpret individual predictions by identifying the most influential laboratory indicators and their directional contributions (positive or negative). For example, elevated GLU and reduced K and APOA1 levels may indicate a high risk of posterior circulation ischemia, thus prompting further diagnostic investigation or tailored interventions. SHAP improves the transparency of machine learning predictions and provides actionable insights for personalized clinical decision-making. To facilitate the usage of our model in clinical settings, we propose developing a web application with this work. This tool would calculate and return the probability of nervous system diseases for individual patients when their relevant feature values are inputted. With this tool, our prediction model can be accessed online and easily shared with healthcare professionals [Identification and validation of an explainable prediction model of acute kidney injury with prognostic implications in critically ill children: a prospective multicenter cohort study]. However, clinical application requires multi-center validation of the model’s effectiveness. The quality of the data determines the reliability of the ML model^50^. Continuing to collect data and increasing the number of patients and blood samples may further enhance the model’s performance.

Our primary future goal is to establish a multi-center collaboration to enhance the diversity and generalizability of our diagnostic models across different clinical settings. Additionally, we aim to integrate advanced machine learning technologies, such as neural networks and ensemble methods, to improve the diagnostic accuracy of our models. Furthermore, we are willing to develop a real-time diagnostic tool using explainable artificial intelligence (XAI), which is expected to help healthcare providers utilize routine blood tests for immediate clinical decision-making^19^. This tool will likely increase the clinical utility of blood tests and improve patient care efficiency. These initiatives will significantly advance our diagnostic framework, potentially offering more precise and cost-effective solutions for the diagnosis of neurological diseases.

This study has the following strengths. Firstly, the data used to construct the model in this research, derived from blood routine and blood biochemical tests, originate from hospital laboratories, making them easy to obtain. This can help doctors in early diagnosis and reduce costs. Consequently, it has the potential for widespread adoption across various healthcare institutions. Secondly, the neurological disorders included are selected based on the ICD-10 codes, encompassing a total of 27 kinds of neurological diseases, making the model applicable to various healthcare institutions. Moreover, we used the SHAP algorithm to explore distinguishing features between patients with neurological disorders and healthy populations, as well as features for differentiation among various types of neurological disorders. This provides important clues for doctors when reviewing laboratory reports. Additionally, these feature indicators can assist patients and public health advisors in understanding the risk factors for neurological disease onset, thus aiding in early disease prevention.

Our study does have some limitations. Primarily, the analysis was conducted using data from a single center, which could limit the generalizability of our findings. Nevertheless, by employing standardized procedures, reagents, and technology that are widely approved, we expect that similar results could be achieved in other settings. This expectation is supported by the robust AUC values observed in our study, indicating a high level of accuracy in disease prediction. These results validate the effectiveness of our model in clinical diagnostic scenarios, even with a constrained data scope. Additionally, the retrospective nature of the study restricted the scope of information obtained from our patients. However, for our purposes, we mainly required results from routine blood tests and accurate diagnoses, which were consistently available for all patients. Owing to its proven ability to distinguish between various neurological conditions effectively, our study establishes a solid foundation for future expansions and applications in more varied clinical environments. To ensure broader applicability and acceptance within the medical community, it is crucial that future studies replicate and validate our findings across multi-center settings.

## Conclusions

The present study constructed multiple models using 52 features from the blood routine and biochemical detection data for diagnosis of various nervous system diseases. Meanwhile, distinct hematologic features of various nervous system diseases also were explored. This can help doctors realize early diagnosis and prevention of nervous system diseases (Supplementary Fig. 5).

## Electronic supplementary material

Below is the link to the electronic supplementary material.

Supplementary Material 1

Supplementary Material 2

## Data Availability

Data cannot be published without patients’ consent. Researchers who are interested for academic need could contact the corresponding authors.

## Abbreviations

DALYs: disability-adjusted life-years
LR: logistic regression
RF: random forest
SVM: support vector machine
XGBoost: extreme gradient boosting
GBDT: gradient boosting decision tree
DNN: deep neural network
NMO: neuromyelitis optica
MRI: magnetic resonance imaging
LY%: lymphocyte percentage
HCT: hematocrit

## References

[1] Global National burden of disorders affecting the nervous system, 1990–2021: a systematic analysis for the global burden of disease study 2021. *Lancet Neurol.* **23**, 344–381. 10.1016/s1474-4422(24)00038-3 (2024). 38493795 10.1016/S1474-4422(24)00038-3 PMC10949203

[2] Global & national burden of neurological disorders. 1990–2016: a systematic analysis for the global burden of disease study 2016. *Lancet Neurol.* **18**, 459–480. 10.1016/s1474-4422(18)30499-x (2019). 30879893 10.1016/S1474-4422(18)30499-X PMC6459001

[3] Huang, Y., Li, Y., Pan, H. & Han, L. Global, regional, and National burden of neurological disorders in 204 countries and territories worldwide. *J. Global Health*. **13**, 04160. 10.7189/jogh.13.04160 (2023). 10.7189/jogh.13.04160 PMC10685084 38018250

[4] Chételat, G. et al. Amyloid-PET and (18)F-FDG-PET in the diagnostic investigation of alzheimer’s disease and other dementias. *Lancet Neurol.* **19**, 951–962. 10.1016/s1474-4422(20)30314-8 (2020). 33098804 10.1016/S1474-4422(20)30314-8

[5] Buchanan, S. M., Richards, M., Schott, J. M. & Schrag, A. Mild parkinsonian signs: A systematic review of clinical, imaging, and pathological associations. *Mov. Disorders: Official J. Mov. Disorder Soc.* **36**, 2481–2493. 10.1002/mds.28777 (2021). 10.1002/mds.28777 34562045

[6] Verger, A., Grimaldi, S., Ribeiro, M. J., Frismand, S. & Guedj, E. Single photon emission computed tomography/positron emission tomography molecular imaging for parkinsonism: A Fast-Developing field. *Ann. Neurol.* **90**, 711–719. 10.1002/ana.26187 (2021). 34338333 10.1002/ana.26187 PMC9291534

[7] Dage, J. L. et al. Cerebrospinal fluid biomarkers in the longitudinal Early-onset alzheimer’s disease study. *Alzheimer’s Dement. J. Alzheimer’s Assoc.* **19** (Suppl 9), S115–s125. 10.1002/alz.13399 (2023). 10.1002/alz.13399 PMC10877673 37491668

[8] Karayel, O. et al. Proteome profiling of cerebrospinal fluid reveals biomarker candidates for parkinson’s disease. *Cell. Rep. Med.* **3**, 100661. 10.1016/j.xcrm.2022.100661 (2022). 35732154 10.1016/j.xcrm.2022.100661 PMC9245058

[9] Gentile, F. et al. The value of routine blood work-up in clinical stratification and prognosis of patients with amyotrophic lateral sclerosis. *J. Neurol.* **271**, 794–803. 10.1007/s00415-023-12015-3 (2024). 37801095 10.1007/s00415-023-12015-3 PMC10827966

[10] Sun, L. et al. Causal associations of blood lipids with risk of ischemic stroke and intracerebral hemorrhage in Chinese adults. *Nat. Med.* **25**, 569–574. 10.1038/s41591-019-0366-x (2019). 30858617 10.1038/s41591-019-0366-x PMC6795549

[11] Novellino, F., Donato, A., Malara, N., Madrigal, J. L. & Donato, G. Complete blood cell count-derived ratios can be useful biomarkers for neurological diseases. *Int. J. ImmunoPathol Pharmacol.* **35**, 20587384211048264. 10.1177/20587384211048264 (2021). 34569352 10.1177/20587384211048264 PMC8477675

[12] Heo, J. et al. Machine Learning-Based model for prediction of outcomes in acute stroke. *Stroke* **50**, 1263–1265. 10.1161/strokeaha.118.024293 (2019). 30890116 10.1161/STROKEAHA.118.024293

[13] Li, X., Wang, Y. & Xu, J. Development of a machine learning-based risk prediction model for cerebral infarction and comparison with nomogram model. *J. Affect. Disord.* **314**, 341–348. 10.1016/j.jad.2022.07.045 (2022). 35882300 10.1016/j.jad.2022.07.045

[14] Tsukita, K. et al. High-Throughput CSF proteomics and machine learning to identify proteomic signatures for Parkinson disease development and progression. *Neurology* **101**, e1434–e1447. 10.1212/wnl.0000000000207725 (2023). 37586882 10.1212/WNL.0000000000207725 PMC10573147

[15] Ning, W. et al. Open resource of clinical data from patients with pneumonia for the prediction of COVID-19 outcomes via deep learning. *Nat. Biomedical Eng.* **4**, 1197–1207. 10.1038/s41551-020-00633-5 (2020). 10.1038/s41551-020-00633-5 PMC7723858 33208927

[16] Yu, Z., Stewart, T., Aasly, J., Shi, M. & Zhang, J. Combining clinical and biofluid markers for early parkinson’s disease detection. *Ann. Clin. Transl. Neurol.* **5**, 109–114. 10.1002/acn3.509 (2018). 29376098 10.1002/acn3.509 PMC5771326

[17] Omrani, M. et al. Machine learning-driven diagnosis of multiple sclerosis from whole blood transcriptomics. *Brain. Behav. Immun.* **121**, 269–277. 10.1016/j.bbi.2024.07.039 (2024). 39097200 10.1016/j.bbi.2024.07.039

[18] Kononikhin, A. S. et al. Prognosis of Alzheimer’s Disease Using Quantitative Mass Spectrometry of Human Blood Plasma Proteins and Machine Learning. *Int. J. Mol. Sci.* **23**10.3390/ijms23147907 (2022). 10.3390/ijms23147907 PMC9318764 35887259

[19] Tasci, B. et al. Automated schizophrenia detection model using blood sample scattergram images and local binary pattern. *Multimedia Tools Appl.* **83**, 42735–42763. 10.1007/s11042-023-16676-0 (2024).

[20] Wang, H. et al. eHSCPr discriminating the cell identity involved in endothelial to hematopoietic transition. *Bioinf. (Oxford England)*. **37**, 2157–2164. 10.1093/bioinformatics/btab071 (2021). 10.1093/bioinformatics/btab071 33532815

[21] Tang, H. et al. HBPred: a tool to identify growth hormone-binding proteins. *Int. J. Biol. Sci.* **14**, 957–964. 10.7150/ijbs.24174 (2018). 29989085 10.7150/ijbs.24174 PMC6036759

[22] Zulfiqar, H. et al. Identification of Cyclin protein using gradient boost decision tree algorithm. *Comput. Struct. Biotechnol. J.* **19**, 4123–4131. 10.1016/j.csbj.2021.07.013 (2021). 34527186 10.1016/j.csbj.2021.07.013 PMC8346528

[23] Zhang, D. et al. iCarPS: a computational tool for identifying protein carbonylation sites by novel encoded features. *Bioinf. (Oxford England)*. **37**, 171–177. 10.1093/bioinformatics/btaa702 (2021). 10.1093/bioinformatics/btaa702 32766811

[24] Kriegeskorte, N. & Golan, T. Neural network models and deep learning. *Curr. Biol. CB*. **29** R231-r236 (2019). 10.1016/j.cub.2019.02.034 30939301

[25] Zhang, L. et al. A deep learning model to identify gene expression level using cobinding transcription factor signals. *Brief. Bioinform.* **23**10.1093/bib/bbab501 (2022). 10.1093/bib/bbab501 34864886

[26] Meng, L. et al. Mini-review: recent advances in post-translational modification site prediction based on deep learning. *Comput. Struct. Biotechnol. J.* **20**, 3522–3532. 10.1016/j.csbj.2022.06.045 (2022). 35860402 10.1016/j.csbj.2022.06.045 PMC9284371

[27] Liu, M. et al. A computational framework of routine test data for the cost-effective chronic disease prediction. *Brief. Bioinform.* **24**10.1093/bib/bbad054 (2023). 10.1093/bib/bbad054 36772998

[28] Wang, K. et al. Interpretable prediction of 3-year all-cause mortality in patients with heart failure caused by coronary heart disease based on machine learning and SHAP. *Comput. Biol. Med.* **137**, 104813. 10.1016/j.compbiomed.2021.104813 (2021). 34481185 10.1016/j.compbiomed.2021.104813

[29] Rajput, A. H. & Rajput, A. Accuracy of Parkinson disease diagnosis unchanged in 2 decades. *Neurology* **83**, 386–387. 10.1212/wnl.0000000000000653 (2014). 24975858 10.1212/WNL.0000000000000653

[30] Porsteinsson, A. P., Isaacson, R. S., Knox, S., Sabbagh, M. N. & Rubino, I. Diagnosis of early alzheimer’s disease: clinical practice in 2021. *J. Prev. Alzheimer’s Disease*. **8**, 371–386. 10.14283/jpad.2021.23 (2021). 34101796 10.14283/jpad.2021.23 PMC12280795

[31] Riveros Gilardi, B. et al. Types of cerebral herniation and their imaging features. *Radiographics: Rev. Publication Radiological Soc. North. Am. Inc*. **39**, 1598–1610. 10.1148/rg.2019190018 (2019). 10.1148/rg.2019190018 31589570

[32] Haller, S., Haacke, E. M., Thurnher, M. M. & Barkhof, F. Susceptibility-weighted imaging: technical essentials and clinical neurologic applications. *Radiology* **299**, 3–26. 10.1148/radiol.2021203071 (2021). 33620291 10.1148/radiol.2021203071

[33] Rustenhoven, J. et al. Functional characterization of the dural sinuses as a neuroimmune interface. *Cell* **184**, 1000–1016. .e1027 (2021). 33508229 10.1016/j.cell.2020.12.040 PMC8487654

[34] Zhang, Z. et al. Abnormal immune function of B lymphocyte in peripheral blood of Parkinson’s disease. *Parkinsonism Relat. Disord.* **116**10.1016/j.parkreldis.2023.105890 (2023). 10.1016/j.parkreldis.2023.105890 37839276

[35] Garfias, S. et al. Peripheral blood lymphocyte phenotypes in alzheimer and parkinson’s diseases. *Neurologia* **37**, 110–121. 10.1016/j.nrleng.2018.10.022 (2022). 10.1016/j.nrleng.2018.10.022 35279225

[36] Sibille, J., Dao Duc, K., Holcman, D. & Rouach, N. The neuroglial potassium cycle during neurotransmission: role of Kir4.1 channels. *PLoS Comput. Biol.* **11**, e1004137. 10.1371/journal.pcbi.1004137 (2015). 25826753 10.1371/journal.pcbi.1004137 PMC4380507

[37] Kelty, T. J., Dashek, R. J., Arnold, W. D. & Rector, R. S. Emerging links between nonalcoholic fatty liver disease and neurodegeneration. *Semin. Liver Dis.* **43**, 77–88. 10.1055/s-0043-1762585 (2023). 36764305 10.1055/s-0043-1762585 PMC12433240

[38] Ma, H. K. & Bebawy, J. F. Albumin use in Brain-injured and neurosurgical patients: concepts, indications, and controversies. *J. Neurosurg. Anesthesiol.* **33**, 293–299. 10.1097/ana.0000000000000674 (2021). 31929351 10.1097/ANA.0000000000000674

[39] Kamada, H., Yu, F., Nito, C. & Chan, P. H. Influence of hyperglycemia on oxidative stress and matrix metalloproteinase-9 activation after focal cerebral ischemia/reperfusion in rats: relation to blood-brain barrier dysfunction. *Stroke* **38**, 1044–1049. 10.1161/01.STR.0000258041.75739.cb (2007). 17272778 10.1161/01.STR.0000258041.75739.cb PMC1828129

[40] Mühlebach, S. M. et al. Sequence homology and structure predictions of the creatine kinase isoenzymes. *Mol. Cell. Biochem.* **133–134**, 245–262. 10.1007/bf01267958 (1994). 10.1007/BF01267958 7808457

[41] Ceccanti, M. et al. Creatine kinase and progression rate in amyotrophic lateral sclerosis. *Cells* **9**10.3390/cells9051174 (2020). 10.3390/cells9051174 PMC7291088 32397320

[42] Li, S. et al. Creatine kinase is associated with recurrent stroke and functional outcomes of ischemic stroke or transient ischemic attack. *J. Am. Heart Assoc.*. **11**, e022279. 10.1161/jaha.121.022279 (2022). 10.1161/JAHA.121.022279 PMC9075278 35243903

[43] Brigo, F. et al. Postictal serum creatine kinase for the differential diagnosis of epileptic seizures and psychogenic non-epileptic seizures: a systematic review. *J. Neurol.* **262**, 251–257. 10.1007/s00415-014-7369-9 (2015). 24824225 10.1007/s00415-014-7369-9

[44] Cochran, B. J., Ong, K. L., Manandhar, B. & Rye, K. A. APOA1: a protein with multiple therapeutic functions. *Curr. Atheroscler. Rep.* **23**10.1007/s11883-021-00906-7 (2021). 10.1007/s11883-021-00906-7 33591433

[45] Bahrami, A., Barreto, G. E., Lombardi, G., Pirro, M. & Sahebkar, A. Emerging roles for high-density lipoproteins in neurodegenerative disorders. *BioFactors (Oxford England)*. **45**, 725–739. 10.1002/biof.1541 (2019). 31301192 10.1002/biof.1541

[46] Xia, Y., Lai, W., Li, S., Wen, Z. & Chen, L. Differentiation of epilepsy and psychogenic nonepileptic events based on body fluid characteristics. *Epilepsia Open.* **8**, 959–968. 10.1002/epi4.12775 (2023). 37329211 10.1002/epi4.12775 PMC10472377

[47] Xia, H. et al. Predicting transient ischemic attack risk in patients with mild carotid stenosis using machine learning and CT radiomics. *Front. Neurol.* **14**10.3389/fneur.2023.1105616 (2023). 10.3389/fneur.2023.1105616 PMC9944715 36846119

[48] Chang, C. C., Liu, T. C., Lu, C. J., Chiu, H. C. & Lin, W. N. Explainable machine learning model for identifying key gut microbes and metabolites biomarkers associated with myasthenia Gravis. *Comput. Struct. Biotechnol. J.* **23**, 1572–1583. 10.1016/j.csbj.2024.04.025 (2024). 38650589 10.1016/j.csbj.2024.04.025 PMC11035017

[49] Lam, S., Arif, M., Song, X., Uhlén, M. & Mardinoglu, A. Machine learning analysis reveals biomarkers for the detection of neurological diseases. *Front. Mol. Neurosci.* **15**, 889728. 10.3389/fnmol.2022.889728 (2022). 35711735 10.3389/fnmol.2022.889728 PMC9194858

[50] Hohmann, E. & Editorial Commentary Big data and machine learning in medicine. *Arthroscopy: J. Arthroscopic Relat. Surg.*. **38**, 848–849. 10.1016/j.arthro.2021.10.008 (2022). 10.1016/j.arthro.2021.10.008 35248233

