# Supplementary material for: Machine learning models based on routine blood and biochemical test data for diagnosis of neurological diseases
Source: Sci Rep. 2025 Jul 30;15:27857. doi: 10.1038/s41598-025-09439-4 (PMC12311004; doi:10.1038/s41598-025-09439-4)
Supplement: Supplementary file 1 — Supplementary Material 1 [file 41598_2025_9439_MOESM1_ESM.docx]

**Supplementary Figures**

**
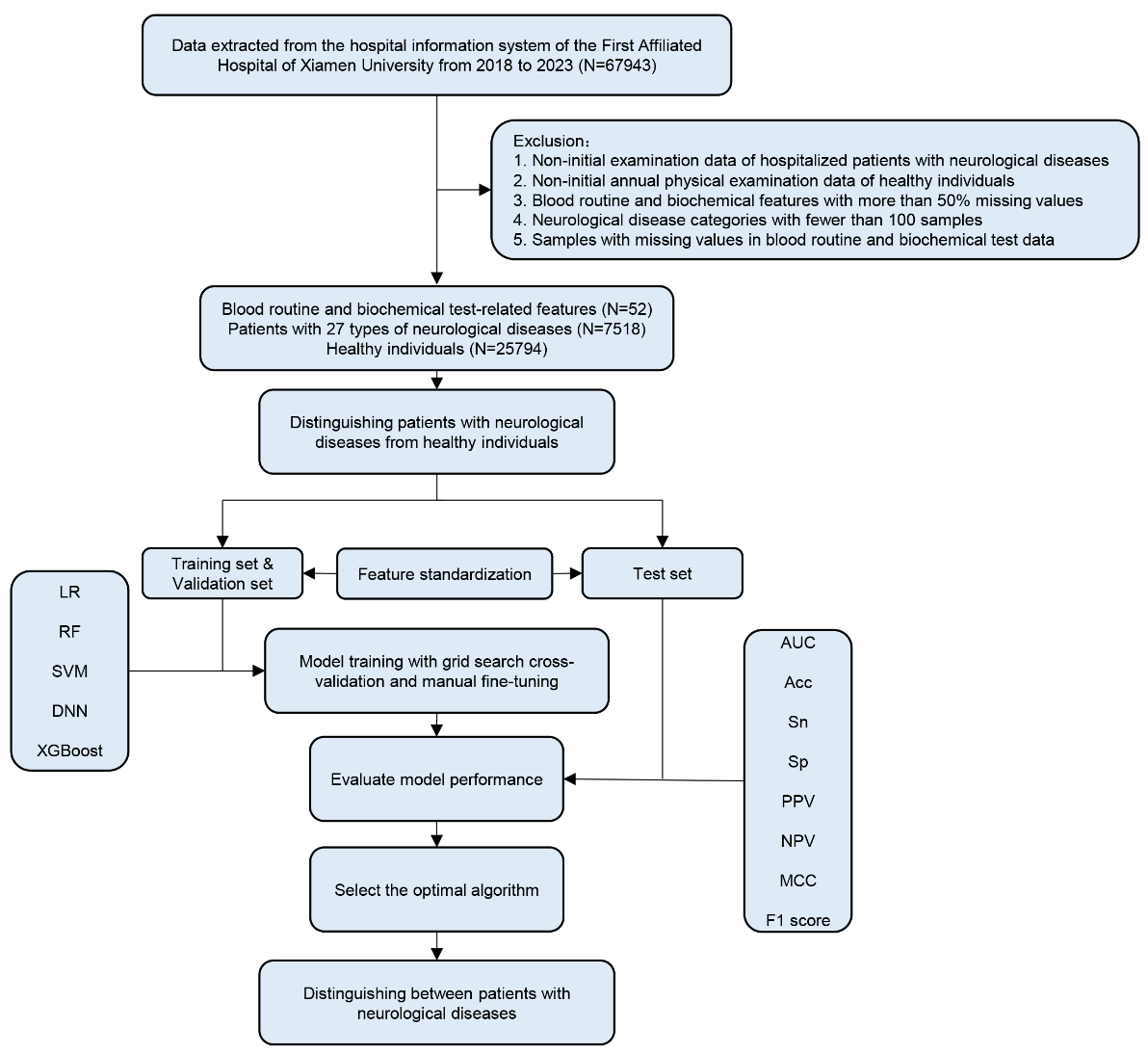
Supplementary Figure 1.** Flowchart from data preprocessing to model evaluation.


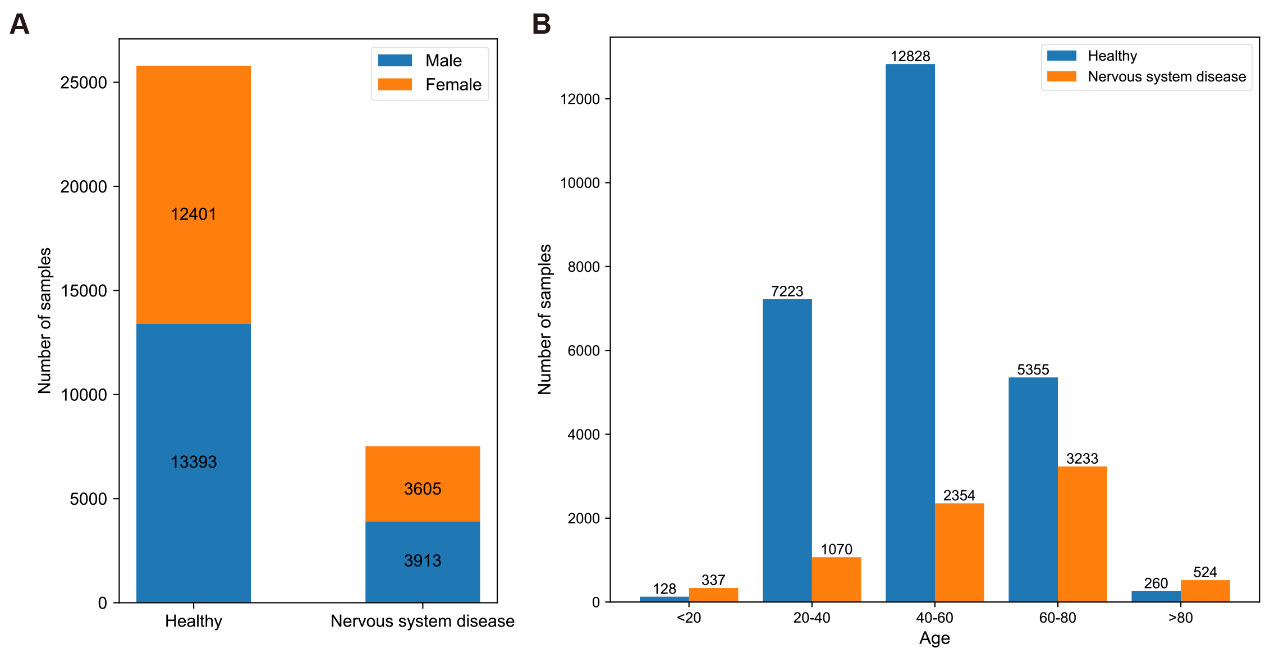
**Supplementary Figure 2.** Statistics of age and gender in healthy people and patients with nervous system disease.

A. Gender distribution of healthy people versus patients with nervous system disease. B. Age distribution of healthy people versus patients with nervous system disease.

**Supplementary Figure 3.** Performance of XGBoost after splitting the dataset by time.


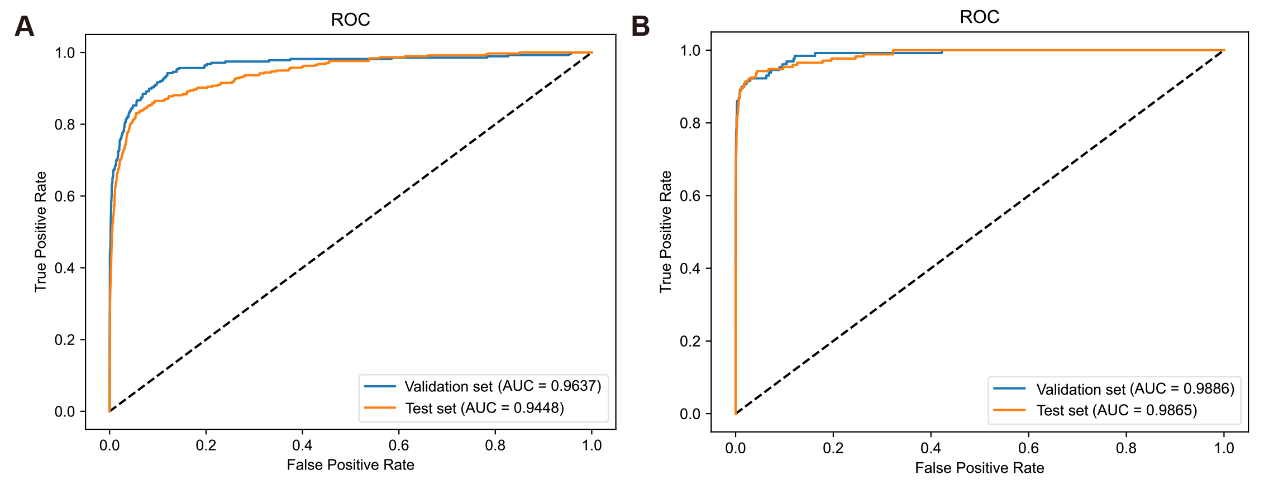


A. Differentiation between post-circulatory ischemia patients and healthy individuals. B. Differentiation between hydrocephalus patients and healthy individuals.

**Supplementary Figure 4.** Performance demonstration using the top 10 features of importance to predict nervous system disease.


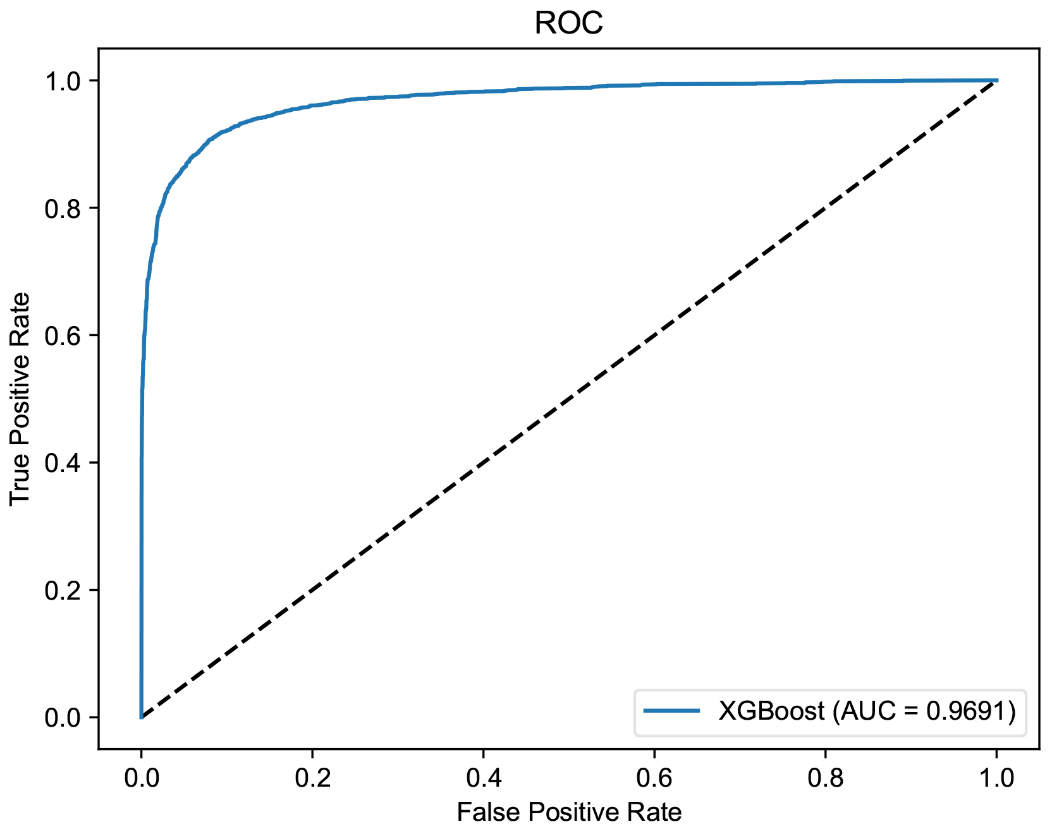


**Supplementary Figure 5.** Classification tree of nervous system diseases.

**
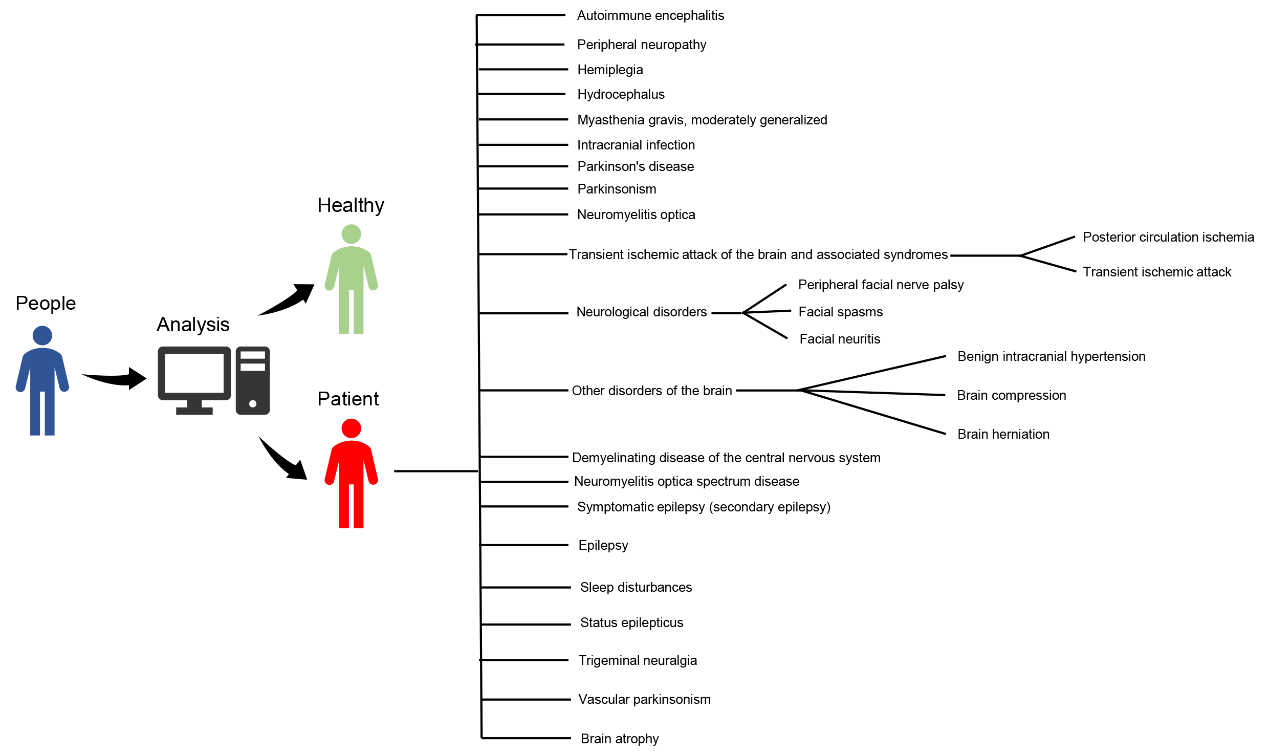
**

**Supplementary Tables**

**Supplementary Table 1.** 22 test indicators of blood routine.

| Feature | Abbreviation | Unit | Reference |
| --- | --- | --- | --- |
| White blood cell count | WBC | ×10^9^/L | 3.5~9.5 |
| Neutrophil percentage | NE% | % | 40~75 |
| Lymphocyte percentage | LY% | % | 20~50 |
| Monocyte percentage | MO% | % | 3~10 |
| Eosinophil percentage | EO% | % | 0.4~8.0 |
| Basophil percentage | BA% | % | 0~1 |
| Absolute value of neutrophil | NE# | ×10^9^/L | 1.8~6.3 |
| Absolute value of lymphocytes | LY# | ×10^9^/L | 1.1~3.2 |
| Absolute value of monocyte | MO# | ×10^9^/L | 0.1~0.6 |
| Absolute value of eosinophil | EO# | ×10^9^/L | 0.02~0.52 |
| Absolute value of basophil | BA# | ×10^9^/L | 0~0.06 |
| Red blood cell count | RBC | ×10^12^/L | 3.8~5.8 |
| Hemoglobin | HGB | g/L | 115~150 |
| Hematocrit | HCT | L/L | 0.35~0.50 |
| Mean corpuscular volume | MCV | fL | 82~100 |
| Mean corpuscular hemoglobin | MCH | pg | 27~34 |
| Mean erythrocyte hemoglobin concentration | MCHC | g/L | 316~354 |
| Red blood cell distribution width | RDW | % | 11~16 |
| Platelet count | PLT | ×10^9^/L | 125~350 |
| Plateletcrit | PCT | % | 0.11~0.27 |
| Mean platelet volume | MPV | fL | 9.4~12.5 |
| Platelet distribution width | PDW | fL | 9~17 |

**Supplementary Table 2.** 30 biochemical detection indicators.

| Feature | Abbreviation | Unit | Reference |
| --- | --- | --- | --- |
| Alanine aminotransferase | ALT | U/L | 7~50 |
| Aspartate aminotransferase | AST | U/L | 13~40 |
| Aspartate aminotransferase/ Alanine aminotransferase | AST/ALT |  | - |
| Alkaline phosphatase | ALP | U/L | 35~150 |
| Gamma-glutamyl Transferase | GGT | U/L | 7~60 |
| Total protein | TP | g/L | 65~85 |
| Albumin | ALB | g/L | 40~55 |
| Globulin | GLO | g/L | 20~40 |
| Albumin/Globulin | A/G |  | (1.2~2.4)：1 |
| Potassium | K | mmol/L | 3.5~5.3 |
| Sodium | Na | mmol/L | 137~147 |
| Chlorine | CL | mmol/L | 99~110 |
| Total bilirubin | TBIL | µmol/L | ≤ 26.0 |
| Direct bilirubin | DBIL | µmol/L | ≤ 4.0 |
| Urea | UREA | mmol/L | 2.6~9.5 |
| Creatinine | CRE | µmol/L | 41~111 |
| Calcium | Ca | mmol/L | 2.11~2.52 |
| Phosphorus | IP | mmol/L | 0.85~1.51 |
| Magnesium | Mg | mmol/L | 0.75~1.02 |
| Lactate dehydrogenase | LDH | U/L | 120~250 |
| Creatine kinase | CK | U/L | 40~310 |
| Cholesterol | CHO | mmol/L | 3.1~5.2 |
| Indirect bilirubin | NBIL | µmol/L | ≤ 22.0 |
| Triglycerides | TG | mmol/L | 0.4~1.82 |
| Uric acid | UA | µmol/L | 155~428 |
| Low-density lipoprotein cholesterol | LDL-C | mmol/L | ≤ 3.1 |
| Glucose | GLU | mmol/L | 3.9~6.1 |
| Apolipoprotein A1 | APOA1 | g/L | 1.0~1.6 |
| Apolipoprotein B | APOB | g/L | 0.6~1.1 |
| Carbon dioxide | CO2 | mmol/L | 21~31 |
